# Chondroitin Sulfate-Based Nanoplatforms: Advances and Challenges for Cancer Therapy

**DOI:** 10.3390/molecules30244798

**Published:** 2025-12-16

**Authors:** Ludovica Scorzafave, Marco Fiore, Giuseppe Cirillo, Fiore Pasquale Nicoletta, Francesca Iemma, Manuela Curcio

**Affiliations:** Department of Pharmacy Health and Nutritional Science, University of Calabria, 87036 Rende, Italy; ludovica.scorzafave@unical.it (L.S.); marcofiore492@gmail.com (M.F.); fiore.nicoletta@unical.it (F.P.N.); francesca.iemma@unical.it (F.I.); manuela.curcio@unical.it (M.C.)

**Keywords:** chondroitin sulfate, smart nanoparticles, drug targeting, cancer therapy

## Abstract

Chondroitin sulfate (CS)-based nanoparticles have emerged as versatile and multifunctional platforms for cancer therapy, integrating effective drug delivery with diagnostic capabilities. Their ability to exploit the enhanced permeability and retention (EPR) effect enables selective accumulation within tumor tissues, while surface modification with CS enhances targeting efficiency through strong conformational and electrostatic affinity for CD44 receptors, which are overexpressed in many cancer cells. In addition, CS interacts with E-selectin, providing dual-targeting capabilities superior to those of other polysaccharides such as hyaluronic acid. A wide variety of CS-derived nanostructures—including micelles, nanogels, hybrid liposomes, and CS–drug conjugates—have shown great potential not only in drug delivery but also in advanced therapeutic modalities such as photodynamic, sonodynamic, and immunotherapy. This review discusses recent advances (2020–2025) in CS-based nanoplatforms for cancer therapy, with particular emphasis on the role of CS within nanostructures. It highlights how the functionalization of nanoparticles with CS represents a powerful strategy to improve colloidal stability, pharmacokinetics, and receptor-mediated uptake, thereby enabling controlled, site-specific drug release and reducing off-target toxicity. Ultimately, these advances open new opportunities for cancer treatment, with the potential for bench-to-clinic translation through the integration of AI-guided design, organelle-specific targeting, multi-pathway modulation, and immune system engagement.

## 1. Introduction

According to World Health Organization statistics for 2022, there were an estimated 20 million new cancer cases and 9.7 million cancer-related deaths worldwide. Approximately 53.5 million people were living within five years of a cancer diagnosis. Overall, about one in five people develop cancer during their lifetime, with roughly one in nine men and one in twelve women dying from the disease [1].

Despite rapid advances in nanotechnology and medical research, cancer treatment remains one of the greatest challenges to human health. Conventional therapeutic approaches, such as chemotherapy and radiotherapy, often fail to achieve optimal outcomes due to severe off-target side effects [2]. Nanotechnology plays a central role in overcoming these limitations, and a wide range of smart nanoplatforms based on pharmaceutical nanotechnology have been developed to enable site-specific delivery, tumor microenvironment responsiveness, and controlled or switchable drug release, while improving safety and stability in biological systems [3,4].

The success of a nanoplatform designed as a drug delivery system (DDS) largely depends on the physicochemical properties of both the nanocarrier and the therapeutic payload [5]. Among the various nanocarrier materials, polymers, whether synthetic or natural, have attracted significant attention for their ability to improve drug pharmacokinetics, prolong systemic circulation, and enhance tissue-specific targeting. In particular, natural biocompatible and biodegradable polymers, such as proteins and polysaccharides, are preferred in pharmaceutical applications because they are non-immunogenic and responsive to physiological stimuli such as pH or temperature variations. Moreover, their biodegradability ensures safe elimination from the body, minimizing accumulation and long-term toxicity [6,7,8].

**Figure 1:**
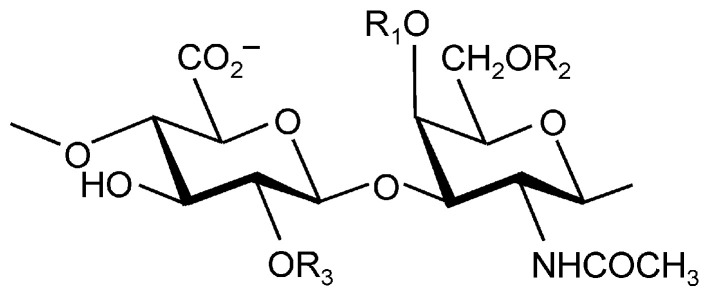
Chemical structure of CS. R1, R2, and R3 represent sites of sulfation. Reproduced from [11].

Among polysaccharides, chondroitin sulfate (CS) has emerged as a particularly promising biopolymer for smart DDS design. CS is a naturally occurring anionic mucopolysaccharide belonging to the glycosaminoglycan (GAG) family, abundantly present in the extracellular matrix, cartilage, bone, skin, nerve tissue, and blood vessels of humans and other species [9]. It consists of alternating copolymers of (β-1,3)-linked glucuronic acid and (β-1,4)-linked N-acetylgalactosamine and exists in several isomeric forms, depending on the number and position of sulfate groups attached to the disaccharide units (Figure 1) [10].

CS displays a wide range of biological and pharmacological activities, many of which stem from its characteristic structural features, particularly chain length and sulfation pattern. Like all polysaccharides, the length of the CS polymer strongly influences its physicochemical behavior, including viscosity, solubility, stability, and protein binding capacity. Longer chains, which confer higher molecular weight and greater viscosity, are essential for functions such as joint lubrication in synovial fluid [12]. Chain length also modulates how CS interacts with proteins, its susceptibility to enzymatic degradation, and its distribution in biological systems, thereby affecting its pharmacokinetic profile in medical applications [13].

Equally important is the diversity of CS sulfation patterns. Variations in both the number and position of sulfate groups on its disaccharide units create distinct regions of high negative charge density [14]. These charged domains play a critical role in shaping CS interactions with water and with surrounding biological components, provide better stealth properties compared to positively charged polysaccharides (e.g., Chitosan/Chitin) [15]. Through these interactions, CS contributes to the assembly and organization of the extracellular matrix (ECM), binding to proteins and proteoglycans to help create a dynamic, highly ordered microenvironment [16]. Sulfation patterns are therefore key determinants of CS bioactivity, influencing protein recognition, cell signaling pathways, tissue development, and the maintenance of tissue homeostasis [17]. These structural properties translate into unique bioactive and therapeutic functions.

Unlike many inert polysaccharide carriers used in drug delivery (e.g., Dextran, Algi-nate, Pectins), CS possesses inherent biological activities that can act synergistically with encapsulated therapeutics [15]. CS exhibits well-documented anti-inflammatory effects, sup-pressing the production of mediators such as phospholipase A_2_, cyclooxygenase-2, nitric oxide synthase-2, and C-reactive protein, while also inhibiting neutrophil phagocytosis and oxidative bursts [12]. In animal models of arthritis, oral CS has been shown to reduce disease severity, oxidative stress, and chronic inflammation, alongside improvements in antioxidant status [18]. CS-based scaffolds further demonstrate anti-inflammatory capacity by lowering Nitroxide (NO) and Prostaglandin PGE_2_ levels and promoting cartilage repair through downregulation of Prostaglandins and Inducible Nitric Oxide Synthase (iNOS) expression [19]. Beyond its anti-inflammatory role, CS also exerts antioxidant effects by scavenging reactive oxygen species, helps promote tissue regeneration as a natural ECM component, and even displays antitumor potential by inhibiting cancer cell proliferation and inducing apoptosis. In the nervous system, CS interacts with fibroblast growth factor-2 to regulate neurogenesis, axon guidance, and synapse formation, while influencing neural cell migration by modulating cell polarity [20]. Cardiovascular models likewise highlight CS therapeutic relevance: treatment markedly reduces atherosclerotic plaque formation and enhances endothelial repair, largely through the inhibition of TNF-α-mediated monocyte activation and cytokine release [21].

Another key advantage of CS over other polysaccharides (e.g., Dextran, Alginate, Pec-tins, and Chitosan) relies on its strong conformational and electrostatic affinity for CD44 receptors, which are overexpressed on chondrocytes and tumor cells. Through hydrogen bonding and electrostatic interactions with basic amino acid residues (lysine and argi-nine) on the receptor surface, CS facilitates receptor-mediated endocytosis and enhances targeting efficiency [22,23]. Furthermore, its interaction with E-selectin provides dual targeting capabilities superior to those of polysaccharides such as hyaluronic acid, which is more susceptible to enzymatic degradation by hyaluronidase [24].

CS exhibits moderate enzymatic stability, allowing for safe application in vivo, but should be considered in the design of biologically oriented materials. CS degrades under extreme pH (acidic/basic) or high heat via hydrolysis or beta-elimination, breaking down into smaller fragments and losing sulfate groups, which affects its function and detection [25].

On the other hand, the application of CS in biomedical fields faces several limitations, including batch-to-batch variability, low enzymatic stability, high production costs, and regulatory challenges. The use of CS extracted from animal sources is further restricted by long cultivation periods, limited raw material availability, concerns over zoonotic cross-contamination, and the presence of potential allergenic residues [9]. Many of these issues can be effectively addressed by adopting non-animal derived CS, with one such form already approved by the U.S. Food and Drug Administration as a food ingredient since 2017 [26]. Although several chemical synthesis strategies, such as post-glycosylation transformation, post-glycosylation oxidation, and various semi-synthetic approaches have been developed, the numerous steps involved (protection, activation, coupling, and deprotection) are time-consuming, environmentally demanding, and unsuitable for large scale industrial production. In contrast, biotechnological synthesis offers several advantages, including mild reaction conditions, environmentally friendly processes, and comparatively lower production costs [27].

## 2. CS Nanoplatforms in Cancer Therapy

As before discussed, nanoparticles (NPs) have demonstrated remarkable potential as multifunctional drug carriers for cancer treatment. They can efficiently deliver both therapeutic agents and imaging molecules to solid tumors owing to their ability to exploit the enhanced permeability and retention (EPR) effect, which arises from the leaky tumor vasculature and poor lymphatic drainage. The targeting specificity and retention of NPs can be further improved through surface modification, a key factor in achieving effective tumor therapy [28,29]. Functionalization of NPs with CS, via either non-covalent or covalent strategies, improves colloidal stability, enhances drug pharmacokinetics (solubility and bioavailability), promotes receptor-mediated uptake, and enables controlled targeted delivery of therapeutic agents, thereby reducing off-target toxicity [30,31].

Depending on the conjugation mechanism and composition, various CS-derived nanostructures have been developed, including polymeric micelles, hybrid liposomes, nanogels, lipid NPs, and CS-drug conjugates [32,33]. The abundance of free carboxyl and sulfate groups and the overall negative charge of CS, indeed, allow it to interact with a variety of substrates, including metal ions, amines, lipid macromolecules, and positively charged polymers (e.g., chitosan), forming CS-based complexes, conjugates, or nanoparticle delivery systems [34,35,36,37]. In addition, CS can be chemically modified through amidation or esterification reactions involving its hydroxyl, carboxyl, or amino groups to conjugate hydrophobic drugs, imaging agents, or other polymers. Such modifications enable the design of amphiphilic copolymers capable of self-assembling into tumor-targeting nanocarriers [38,39]. Beyond drug delivery, CS-based systems have shown potential in advanced therapeutic applications, including photodynamic therapy, sonodynamic therapy, immunotherapy, radiotherapy, and magnetic therapy [40].

**Figure 2:**
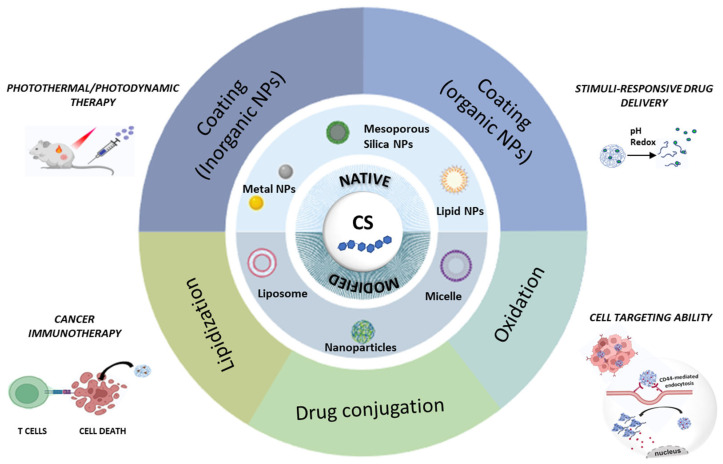
Schematic representation of CS-based nanoplatform for cancer therapy discussed in this review.

This review aims to highlight recent research advances (2020–2025) in CS-based nanoplatforms for cancer therapy, with particular focus on the role of CS within nanostructures. Specifically, we examine cases where CS is employed as a coating material for either organic or inorganic NPs through electrostatic or covalent interactions to impart targeting capability, as well as instances where CS becomes an integral component of the nanoparticle structure through electrostatic forces or following chemical modification that imparts self-assembling properties (Figure 2). Finally, we discuss the design and synthesis of NPs based on CS-drug conjugates. In all cases, we summarize key findings, emerging opportunities, and current challenges in the field.

## 3. CS as a Coating of Organic Nanoparticles

The high affinity of CS for CD44 receptors is the rationale behind its use as a coating material for preformed NPs, achieved either through electrostatic complexation between its negatively charged groups and positively charged nanostructures, or via covalent linkage to specific functional groups on the NP surface.

### 3.1. Coating of Organic NPs

The most recent examples of using CS as a coating element for organic NPs are shown in Table 1.

Nagy and colleagues [41] developed N,N,N-trimethyl chitosan (TMCH) NPs with varying degrees of trimethylation (23–99%) and molecular weights (66–290 kDa), which were subsequently coated with CS via polyelectrolyte complexation (TMCH/CS ratio of 1/1 by weight) to produce biocompatible monodispersed NPs with hydrodynamic diameter of 188 nm. Cytotoxicity studies on ovarian cancer cell lines (SKOV-3 and OVISE) demonstrated a higher cytotoxic effect of TMCH against cancer cells compared to healthy cells (human umbilical vein endothelial cells, HUVECs).

The electrostatic interaction between positively charged doxorubicin (DOX) and negatively charged CS, together with the strong affinity between glycosaminoglycans and proteins, was exploited to design CS-mediated albumin corona NPs that enhanced the cytotoxic activity of the vectorized drug. In vivo studies demonstrated a marked reduction in tumor volume—approximately 50% with free DOX and 80% with DOX-loaded nanoparticles—highlighting the synergistic therapeutic effect of the formulation. The underlying rationale of this approach is that, following intravenous administration, the nanoparticles bind to the gp60 receptor on tumor vascular endothelial cells, thereby activating caveolin-1 and promoting their transcytosis across the endothelium. Subsequent interactions with SPARC and CD44 receptors facilitate nanoparticle accumulation within the tumor interstitium and efficient internalization by cancer cells [42].

CS was also proposed as a coating component for a β-cyclodextrin–polyethylenimine nanosystem (βCyD-PEI) designed for the co-delivery of a small interfering RNA (siRNA) and the chemotherapeutic drug paclitaxel (PTX) [43]. PTX was incorporated via host–guest interactions within βCyD, while siRNA was loaded through electrostatic binding. The βCyD-PEI was incubated with siRNA at different Nitrogen to Phosphorus (N/P) ratios, and Dynamic Light Scattering analyses identified an N/P ratio of 30 as optimal for the subsequent self-assembly between positively charged βCyD-PEI–siRNA and negatively charged CS, yielding NPs with the lowest polydispersity index (PDI = 0.21). The resulting nanocomplexes exhibited strong active targeting toward CD44-overexpressing breast cancer cells. The dual delivery system allows PTX to inhibit tumor cell proliferation, while siRNA targeting CD146 (siCD146) suppresses metastasis and induces apoptosis. Compared to monotherapies, this co-delivery system significantly enhances both cytotoxic and anti-metastatic effects in MDA-MB-231 breast cancer cells, with the IC_50_ (0.16 μg·mL^−1^) decreasing 3.6-fold compared to free PTX and 2-fold compared to control NPs prepared without siRNA.

Curcumin (CUR) is a well-known natural compound with promising anticancer potential, showing excellent in vitro efficacy; however, its clinical application is hindered by poor bioavailability [62]. To overcome this limitation, CUR was incorporated into hydrogels composed of chitosan (CH) and CS, prepared by polyelectrolytic complexation (PEC) using ionic liquids ([Hmim][HSO_4_]) to solubilize the high-molecular-weight CH polysaccharide [44]. CUR entrapment was nearly complete (~100%), and the resulting hydrogels exhibited favorable physicochemical properties, including hydrophobicity, pH-dependent swelling, and controlled dissolution/degradation.

In a complementary approach, CUR was combined with green-synthesized silver NPs (AgNPs) and subjected to visible-light irradiation via Photodynamic Therapy (PDT), which triggered selective cancer cell death through the Metal-Enhanced Singlet Oxygen effect [45]. Cellular assays confirmed that the PEC matrix was non-toxic to healthy tissues, while CUR loaded hydrogels showed high anticancer activity towards Cervix (IC_50_ 327 μg mL^−1^), Colon (IC_50_ 336 μg mL^−1^), and Prostate (IC_50_ 442 μg mL^−1^) cancer cells. Moreover, PDT-mediated selective irradiation of AgNP-loaded hydrogels effectively inhibited the growth of Caco-2 human colon cancer cells with high selectivity. Moreover, cellular uptake studies demonstrated that CUR could function as a theranostic agent, combining therapeutic efficacy with diagnostic fluorescence imaging. Notably, hydrogels prepared in the absence of the ionic liquid were less effective, likely due to the reduced incorporation of CH within the hydrogel network.

Other phytochemicals have also been explored as potent, versatile, and more tolerable cytotoxic agents. Fisetin (Fis), a bioactive flavonol (3,3′,4′,7-tetrahydroxyflavone), although less well-known than other flavonoids, is one of the major polyphenolic compounds naturally occurring in strawberries. It exerts a broad spectrum of biological functions, including antimicrobial, antioxidant, anti-inflammatory, neuroprotective, and anticancer activities. Fis has been shown to inhibit tumor growth by modulating the cell cycle, inducing apoptosis, and suppressing angiogenesis, invasion, and metastasis, while sparing normal cells from toxicity [63]. Pterostilbene (PTS; trans-3,5-dimethoxy-4′-hydroxystilbene), a natural dimethyl ether analogue of resveratrol found in blueberries and grapes, has also demonstrated strong anticancer potential. Its mechanisms of action include inhibition of proliferation, metastasis, invasion, and angiogenesis, as well as suppression of cancer stem cells, induction of apoptosis, and cell cycle arrest [64]. However, similar to curcumin (CUR), the therapeutic applications of both Fis and PTS are limited by poor aqueous solubility, low bioavailability, and photochemical instability, thus necessitating the development of advanced nanocarriers for improved delivery.

To address these limitations, in separate research work, negatively charged phospholipid-stabilized emulsions mimicking natural lipoproteins and solid lipid nanoparticles (SLNs) were used to encapsulate Fis and PTS, respectively [46,47]. These carriers were then subjected to electrostatic layer-by-layer coating, first with a positively charged layer of lactoferrin (Lf) and subsequently with a negatively charged CS layer, both serving as targeting moieties. The resulting nanoparticles exhibited small particle sizes (209 nm for Fis@NPs and 223 nm for PTS@NPs) and negative zeta potentials (−37 mV and −12 mV, respectively). Both NPs effectively controlled and extended the release of their payloads, with Fis@NPs showing a marked improvement in solubility, achieving 100% release after 6 h, and PTS@NPs displaying prolonged release, with 73% release after 24 h. Cytotoxicity assays on breast cancer cells, along with in vivo studies, confirmed a significant enhancement in the anticancer activity of both phytochemicals, with approximately fourfold and threefold reductions in IC_50_ values and corresponding reductions in tumor growth compared to free Fis and PTS, respectively.

A nanotheranostic platform for osteosarcoma was developed by integrating carbon quantum dots (CQDs) as imaging nanoprobes with the cytotoxic drugs DOX and docetaxel (DTX) into chitosan(CH)/CS multilayered nanoparticles, where CQDs served as the hydrophobic core [48]. The nanoparticles exhibited sustained drug release (28 days) and showed strong inhibitory effects against osteosarcoma U2OS and Saos-2 cells, primarily through synergistic drug activity and necrosis-induced cell death.

CS-based nanocarriers were also developed by combining CS derivatives with hydrophobic proteins such as prolamins, plant storage proteins often employed as O/W stabilizers [65]. Among these, foxtail millet prolamin (FMP) and zein (ZN) were proposed for the vectorization of different anticancer drugs through CS coating. In one approach, CUR was efficiently loaded into FMP NPs prepared by solvent displacement in the presence of a sodium caseinate/CS mixture as a stabilizing coating, yielding a nanoplatform with an average size of ~140 nm and a zeta potential of −40 mV. Biological evaluation highlighted the key role of the CS coating, since NPs prepared in the absence of CS showed lower efficacy than the free flavonoid [49]. Moreover, hybrid CS/zein nanoparticles (NPs), prepared in the same manner, were proposed as carriers for docetaxel (DTX) and teriflunomide (TFM) for the treatment of prostate and breast cancer, respectively [50,51]. In vitro studies demonstrated that CS enhanced the colloidal stability and cellular uptake efficiency of zein NPs, as well as improved the pharmacokinetic profiles, with terminal half-lives 1.7- and 9.5-fold longer than those of free TFM and DTX, respectively. NPs exhibited CD44-mediated uptake, as evidenced by reduced internalization following CD44 receptor blocking (1.7- and 0.8-fold decreases in breast and prostate cancer cells, respectively). Compared with the free drugs, the NPs induced higher cytotoxicity and apoptosis, promoted ROS generation, and inhibited cell invasion.

CS coating was employed to enhance the efficiency of dual-responsive (pH- and redox- sensitive) nanoparticles synthesized via the self-assembly of redox responsive PEG–PLA copolymers (PEG–ss–PLA). In one design, the PEG–PLA copolymer was functionalized with thiocollagenase via a maleimide linker to degrade the dense collagen barrier in tumor tissues, facilitated by CS removal under the acidic tumor environment. In vivo studies in breast cancer-bearing mice confirmed the therapeutic potential of these dual pH- and GSH-responsive NPs, which exhibited effective tumor matrix degradation, deep tumor penetration, selective cellular uptake, and the ability to reduce cancer cell proliferation while enhancing apoptosis. These latter effects were demonstrated by the significant decrease in Ki-67–positive tumor areas, from 12.3% with free DOX to 2.1% with the NPs, and by the corresponding increase in TUNEL fluorescence intensity, from 12.6% to 34.8% [52].

In an alternative strategy, triphenylphosphonium (TPP) was grafted onto the PEG–PLA core, and CS served a dual purpose: achieving CD44-mediated targeting and prolonging blood circulation by masking the positive surface charge of TPP. Upon internalization, the acidic endosomal/lysosomal environment triggered CS detachment, exposing positively charged TPP residues, which facilitated mitochondrial localization. This induced membrane depolarization, increased permeability, and excessive reactive oxygen species (ROS) generation, thereby promoting apoptosis. Concurrently, DOX released from the disassembling nanoparticles (84% vs. 30% in physiological conditions) entered the mitochondria, causing mitochondrial DNA damage and enhancing cytotoxicity [53].

The same mechanism was exploited by preparing TPP-D-α-tocopherol polyethylene glycol 1000 succinate- poly(lactide-co-glycolide) NPs (TPP-TPGS-PLGA NPs) loaded with Celastrol (Cls) and coated with a CS-Folic acid (CS-FA) derivative as dual targeting element. Cls is then selectively released in the alkaline mitochondrial environment (pH ~8.0), minimizing premature release in the cytoplasm (pH 7.4) or lysosome (pH 5.0) [54].

The formation of positive charges on the NPs surface after cellular internalization to achieve mitochondrial targeting was also exploited to design of a dual-functionalized lipid–albumin nanosystem for the targeted delivery of paclitaxel (PTX) and the reversal of multidrug resistance (MDR) in breast cancer therapy. NPs, with a hydrodynamic diameter of 176 nm and a zeta potential of −18 mV, were constructed using TPGS as a P-glycoprotein (P-gp) inhibitor to modify cationic liposome structures loaded with a bovine serum albumin (BSA)-PTX complex and subsequently coated with CS. Once internalized into MCF-7/MDR breast cancer cells, the CS layer is predominantly degraded by hyaluronidase, leading to the exposure of the underlying cationic nanosystem. Within mitochondria, the released TPGS disrupts mitochondrial function and inhibits P-gp-mediated drug efflux, thereby enhancing the intracellular accumulation of PTX. In vivo studies confirmed the therapeutic potential of this approach, demonstrating prolonged blood circulation, increased tumor accumulation of PTX, and a tumor inhibition rate of 75.3% [55].

Mitochondrial localization was also exploited as a central design principle in the preparation of CS-coated liposome for the co-delivery of Berberine (Ber) and Magnolol (Mag) (optimal molar ratio 2:1) as natural bioactive ingredients, to lung cancer cells. The nanosystem demonstrated sustained drug release and enhanced uptake by A549 cells, leading to increased cytotoxicity, inhibition of tumor cell migration, and promotion of apoptosis by the regulation of Bcl-2, Bax, and Caspase-3 expression. In addition, in vivo biodistribution and pharmacokinetic studies showed improved blood stability, prolonged circulation time, and enhanced targeting ability, achieving a maximum tumor inhibition rate of 81.48% [56].

In a similar approach, a Ber derivative, 9-O-octadecyl–substituted berberine, serving as a mitochondrial function inhibitor was self-assembled with Hemin, a BACH1 inhibitor, and further coated with CS. The BACH1 targeting reduces the expression of tumor-associated metabolites, glycolytic enzymes, and metastasis-related proteins, while mitochondrial targeting induces membrane depolarization, ROS overproduction, and activation of caspase-3 and caspase-9. These synergistic effects result in the inhibition of tumor cell proliferation, migration, and invasion, ultimately triggering apoptosis. In xenograft mouse models, the treatment achieved a tumor inhibition rate of up to 75%, without significant toxicity to major organs [57].

**Figure 3:**
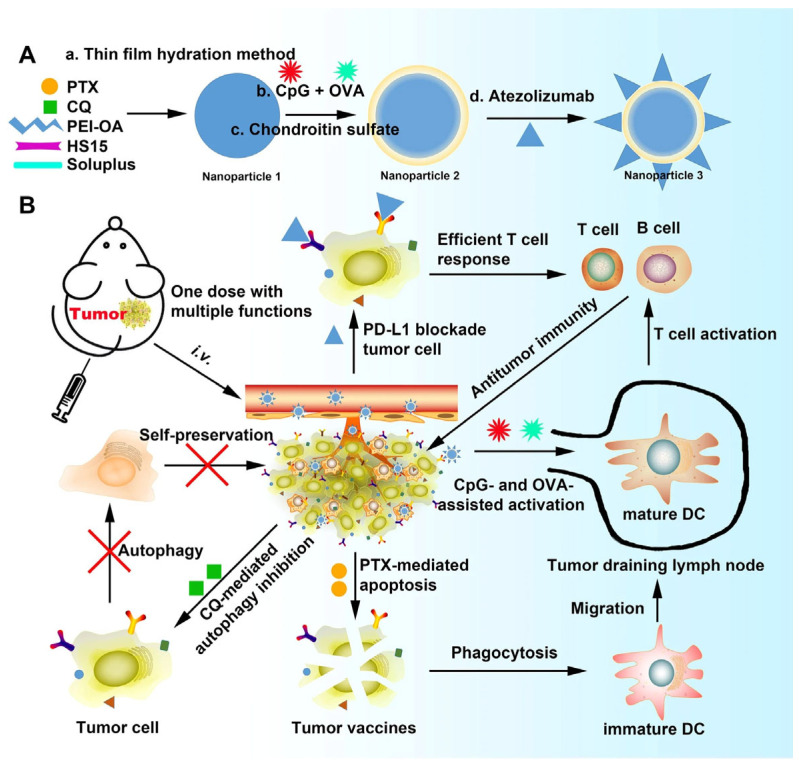
Schematic illustration of (**A**) NPs preparation method, and (**B**) combined application of chemotherapy, immunotherapy, and PD-L1 blockade therapy in tumor-bearing mice. Reproduced from [58].

Multifunctional nanoparticles (NPs) integrating active tumor targeting, autophagy inhibition, chemotherapy, and immune checkpoint blockade were constructed using a PEI–oleic acid (PEI-OA) complex as the core and CS as the shell. The NPs were co-loaded with paclitaxel (PTX), chloroquine (CQ), CpG oligodeoxynucleotides, ovalbumin (OVA), and Atezolizumab (ATZ, an anti-PD-L1 antibody) to enhance tumor targeting and immune activation. Anti-PD-L1 facilitated immune checkpoint blockade and tumor-specific uptake, while CQ reversed the immunosuppressive tumor microenvironment, promoting T-cell infiltration. This combinatorial strategy enhanced cytotoxicity against 4T1 breast cancer cells, increased CD4^+^ and CD8^+^ T-cell infiltration, induced immune memory, inhibited lung metastasis, and achieved sustained antitumor efficacy with the tumor volume reduced up to 70% (Figure 3) [58].

The same PEI-OA complex was used as core element for the preparation of targeted NPs for the co-delivery of CRISPR/Cas9 ribonucleoprotein (RNP) and PTX with the aim of achieving synergistic gene therapy and chemotherapy in hepatocellular carcinoma. The nanoplatform was engineered using a linoleic acid-PEG-octreotide conjugate (LNA-PEG-OCT) to enable somatostatin receptor-mediated targeting of hepatoma cells, and CS coating to improve stability and biocompatibility. The multifunctional system allows for the targeted delivery of both PTX and Cas9 RNP directly to cancer cells, ensuring efficient lysosomal escape and nuclear transport of the CRISPR/Cas9 complex. As a result, high gene-editing efficiency of the PD-L1 gene was achieved, reaching 56% in vitro in HepG2 cells and 46.0% in vivo in tumor tissues. Therapeutically, this led to strong downregulation of PD-L1 protein, which reduced tumor immune escape, and enhanced infiltration of CD8^+^ and CD4^+^ T cells at the tumor site. The treatment also significantly inhibited tumor cell proliferation and achieved an 80% tumor growth suppression in xenograft mouse model, all while maintaining minimal systemic toxicity and demonstrating high liver-targeting efficiency [59].

Chemoimmunotherapy was also the core concept for the development of a liposomal nanoplatform co-loaded with PTX and cryptotanshinone (CTS) and coated with CS and LyP-1 peptide to engage the p32 receptor, enhancing cell-specific uptake. Once internalized, PTX induces immunogenic cell death, promoting cytotoxic T cell infiltration, while CTS suppresses STAT3 signaling, reducing the presence of regulatory T cells, M2-type tumor-associated macrophages, and myeloid-derived suppressor cells. Together, these actions synergistically reverse the immunosuppressive tumor microenvironment, enhancing the overall therapeutic effect [60].

A combinatorial nanotherapeutic strategy was designed to target the immunosuppressive tumor microenvironment, employing nanoparticles co-loaded with the BRD4 inhibitor JQ1 and the COX-2 inhibitor celecoxib (CXB) and coated with CS. These were administered alongside a biomimetic system consisting of poly(β-amino ester)-based nanoparticles coated with melittin-embedded leukocyte membranes. While the use of two separate systems presents challenges for clinical translation, the combination therapy demonstrated synergistic cytotoxicity and enhanced immune activation in TNBC models. Tumor tissue from the combination treatment group exhibited the most pronounced necrotic features, the lowest number of proliferating tumor cells, and the lowest mean microvessel density. Furthermore, the proportion of memory T cells (CD3^+^ CD44^+^ CD62L^−^) in the spleens was significantly increased, indicating effective activation of the antitumor immune response [61].

### 3.2. Coating of Inorganic NPs

Inorganic NPs are emerging as front runners in overcoming several limitations of conventional medicine, owing to their unique optical, electrical, catalytic, and magnetic properties that are not typically observed in organic nanoparticles. Owing to their inherent optical properties, inorganic NPs, either alone or in combination with other imaging agents, are increasingly effective in molecular imaging. In addition, they can be guided to target sites through external stimuli such as light or magnetic fields, offering precise spatial control in therapeutic applications. The versatility of inorganic NPs enables their functionalization with ligands, polymers, and biomolecules, significantly enhancing drug delivery performance. Furthermore, the loading of therapeutic agents such as drugs, nucleic acids, and photosensitizers improves the efficacy of various treatment modalities [66]. The most recent examples of using CS as a coating element for inorganic NPs are collected in Table 2.

Liang et al. [67] proposed CS as a coating for calcium carbonate NPs, a widely studied inorganic material showing biological inertness and ability to dissociate under the acidic environments of the tumor tissues to accelerate the release of the encapsulated drugs. The resulting NPs (~100 nm in size) demonstrated enhanced accumulation in CD44-overexpressing lung cancer cells, improved cellular uptake, and stronger anticancer effects via apoptosis induction (downregulation of the anti-apoptosis factor Bcl-2 and upregulation pro-apoptosis factor Bax) both in vitro and in vivo.

CS was used as a targeting element for nanoscale hydroxyapatite (HAP). It is well known that HAP can induce Ca^2+^ overload, which in turn leads to severe cytotoxicity, since elevated Ca^2+^ levels result in massive oxidative stress and oxidation of mitochondrial DNA. The release of oxidized mitochondrial DNA into the cytoplasm activates the NLRP3 inflammasome, triggering caspase-1 cleavage and Gasdermin activation with the formation of membrane pores, ultimately causing cell swelling, membrane rupture, and the release of pro-inflammatory mediators (IL-1β, IL-18). This represents a potential strategy to reprogram immunosuppressive “cold” tumors into immunogenic “hot” tumors, enriched in tumor-infiltrating lymphocytes [79].

Moreover, bioactive species such as Kaempferol (KAE) and Atorvastatin (ATR) are able to promote Ca^2+^ uptake within cells and mitochondria, respectively, and have thus been investigated for enhancing the anticancer efficacy of PEGylated HAP nanoparticles [68,69].

Mesoporous silica nanoparticles (MSNs) were also conjugated with CS via a cystamine (cys) linker to combine their intrinsic properties (e.g., easily tunable morphology and particle size, hydrophilicity, and surface modifiability) with the targeting activity of CS. CS-MSNs were co-loaded with PTX and quercetin (QR) to overcome MDR in breast cancer. NPs (~227 nm) exhibited redox-responsive drug release and actively target CD44 receptors on MCF-7/ADR cells, enhancing cellular uptake. QR effectively downregulated P-gp expression, reducing drug efflux and increasing PTX retention within resistant tumor cells. This leads to significant G2/M phase cell cycle arrest, microtubule disruption, and increased apoptosis. In vivo, NPs demonstrated prolonged tumor retention and effectively reduced tumor volume while causing minimal toxicity to normal tissues [70].

The need of overcoming P-gp mediated MDR and heat shock proteins (HSPs)-mediated phototherapy resistance motivated the design of a CS-functionalized zeolitic imidazolate framework-8 (ZIF-8) nanoplatform for the co-delivery of DOX and IR780 iodide (IR780)-conjugated atovaquone (ATO) as chemotherapeutic and photosensitizing agents, respectively. In the acidic tumor microenvironment, the released ATO and zinc ions (from ZIF-8) disrupt mitochondrial electron transport and glycolysis through ROS production and inhibition of Glucose transporter 1, leading to energy depletion. This energy exhaustion suppressed P-gp and HSPs, effectively reversing resistance mechanisms [71].

Hydrogen peroxide (H_2_O_2_)/ultrasound-driven mesoporous manganese oxide-based nanomotors were developed by loading indocyanine green (ICG) as mitochondrial sonosensitizers into mesoporous channels and dual-functionalizing their surface with silk fibroin (SF) and CS. The obtained NPs exhibited a desirable size (200 nm), uniform size distribution, with a good colloidal stability due to the negatively charged surface (−27 mV). Endogenous (H_2_O_2_) and exogenous (ultrasound) stimuli induced enhanced NPs locomotory activity, efficient mucus-traversing ability, and deep tumor penetration. In the tumor microenvironment, Mn^2+^ ions catalyzed a Fenton-like reaction with the decomposition of excess H_2_O_2_ into hydroxyl radicals and O_2_. The generated O_2_ further participated in sonodynamic therapy, producing abundant singlet oxygen. Moreover, after cell internalization, ICG is released in response to multiple stimuli, including hydrogen ions, ROS, GSH and ultrasonication. The combined chemodynamic and sonodynamic therapy synergistically induced ferroptosis of tumor cells, promoted the release of tumor antigens, and triggered adaptive immune responses. In vivo experiments demonstrated that oral administration of NPs embedded in a chitosan/alginate hydrogel, in combination with PD-L1 immune checkpoint inhibitors, effectively suppressed both primary and distant tumors [72].

Metal NPs, especially those composed of noble metals such as gold, silver, and palladium, have attracted significant attention in the biomedical field due to their tunable physicochemical properties (e.g., magnetic, optical, and electronic), their high surface area-to-volume ratio, and their capacity for controlled surface functionalization [80].

Metal–organic framework (MOF) materials, organic–inorganic nanohybrids formed by the coordination between metal ions and organic ligands, were also used as a core for CS coating in anticancer therapeutic approaches [81]. A CS coated nanosystem integrated ellagic acid (EA) coordinated with Cu^2+^ to form nanoscale metal–organic frameworks, which are loaded with DOX. EA suppresses tumor cell stemness by inhibiting the hedgehog pathway, while Cu^2+^ disrupts mitochondrial metabolism and induces cuproptosis. This dual action reduces ATP levels, suppresses P-gp activity, and thereby decreases drug efflux, enhancing DOX retention and efficacy in resistant cancer cells [73].

**Figure 4:**
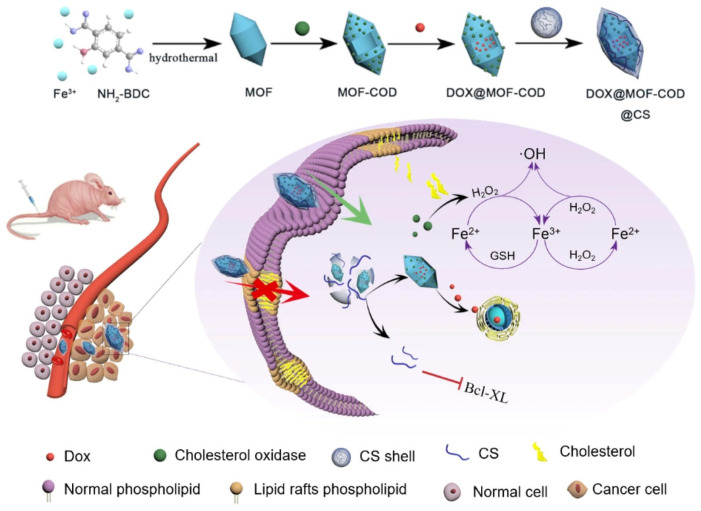
Preparation of nanocarriers based on cascade catalysis and tumor region specific targeting for efficient delivery and reversal of multidrug resistance. Reproduced from [74].

P-gp was also inhibited by CS-coated MOFs immobilizing cholesterol oxidase (COD), which catalyzes cholesterol consumption and generates hydrogen peroxide (H_2_O_2_). The subsequent peroxidase-like activity converts H_2_O_2_ into highly toxic hydroxyl radicals (•OH), thereby inducing cancer cell apoptosis and reversing drug resistance. In vivo, this strategy led to a marked reduction in P-gp activity, with DOX efflux decreasing from 54% for free DOX to 28% for DOX-loaded MOFs (Figure 4) [74].

Gold nanoparticles (AuNPs) are particularly noteworthy, with a long-established record of use in medical applications (e.g., antibacterial, anticancer, antifungal, and wound-healing), owing to their high stability, excellent biocompatibility, low reactogenicity, minimal toxicity both in vitro and in vivo, as well as their distinctive optical and electronic properties, including scattering and absorption capabilities [82]. Mardhekar et al. used spherical gold nanoparticles with a diameter of 20 nm to design glycosaminoglycan (GAG) nanoprobes for studying carbohydrate-mediated cell targeting, demonstrating that CS-coated nanoprobes preferentially target CD44-expressing cancer cells and neurons through caveolin-mediated endocytosis [75].

A green synthesis approach was employed to fabricate spherical AuNPs with an average size of 41 nm, using shark-derived CS as both a stabilizing and targeting agent, in the presence of sodium borohydride as a reducing agent. The resulting CS-AuNPs exhibited significant cytotoxicity against human osteosarcoma cells MG63 at 10 μg mL^−1^, while maintaining excellent biocompatibility with normal mouse fibroblast cells, even at higher concentrations (100 μg mL^−1^). Mechanistic investigations revealed that NPs induced apoptosis in MG63 cells through increased ROS production and mitochondrial dysfunction [76]. In a similar approach, yellow-nose skate (*Dipturus chilensis*) cartilage extract, rich in CS and collagen type II, was employed as both a reducing and stabilizing agent. The resulting nanosystem (c-sk AuNPs), loaded with DOX (loading efficiency of 90%), exhibited an average size of 15 nm and demonstrated the strongest cytotoxicity against breast cancer cells compared with other cell lines (e.g., stomach cancer cells), attributed to their higher uptake efficiency (30 vs. 15%) [77].

CS was also used for the coating of Selenium nanoparticles (Se NPs), which have attracted considerable attention due to their remarkable bioactivity and antioxidant properties. The coated NPs exhibited stronger anti-proliferative effects than unmodified Se NPs in a dose- and time-dependent manner. Mechanistic studies revealed that they induced S-phase cell cycle arrest by downregulating cyclin A and CDK2 while upregulating p21. Apoptosis was triggered through both mitochondrial and death receptor pathways, as evidenced by increased Bax, decreased Bcl-2, cytochrome C release, and activation of caspases-3, -8, and -9. Additionally, they elevated intracellular ROS levels, which likely acted upstream of both S-phase arrest and apoptosis [78].

## 4. Chemical Modification of CS

The chemical modification of CS was also explored as a strategy for preparing targeted NPs via surface coating or for conferring self-assembling properties.

### 4.1. CS Derivatives for NPs Coating

Table 3 summarizes the most relevant examples of CS derivatives proposed as a coating of NPs.

Oxidized CS was used as a gelling agent for the in situ formation of injectable hydrogels through the reaction of OCS aldehyde groups with NH_2_-rich molecules and the formation of reversible Schiff base bonds. Some examples of this approach have been proposed for the post-operative treatment of osteosarcoma and liver cancer, by combining OCS with Gelatin (Gel) and polyacrylamide (PAAm), respectively.

In detail, the osteosarcoma treatment involved the preparation of a mesoporous bioactive glass nanoparticle (MBGN)-MTX conjugate through a serial amidation reaction among aminated MBGN, the redox sensitizer dithiodiglycolic acid, and MTX. This conjugate was then mixed with OCS and allowed to react with Gel to form hydrogel systems. This hydrogel offers dual-stage functionality: in the first phase, the acidic and reductive tumor microenvironment triggers the cleavage of Schiff base bonds, releasing MTX-loaded nanoparticles via pH- and GSH-responsive mechanisms. This release induces ROS generation, mitochondrial dysfunction, and activation of the p16-CYLD tumor suppressor pathway, thereby promoting apoptosis and reducing metastasis. In the later phase, the hydrogel transitions into an osteoconductive scaffold, supporting bone regeneration through enhanced osteogenic differentiation. In vivo studies demonstrated effective suppression of tumors and lung metastasis, along with the promotion of bone repair and minimal systemic toxicity [83].

Regarding liver cancer, a PAAm-OCS hydrogel was loaded with MOFs encapsulating a triple-drug cocktail consisting of the autophagy inhibitor 3-methyladenine (3-MA), the CD73 inhibitor ARL 67,156 trisodium (ARL), and the NETs lyase DNase I. The powder formed an adhesive hydrogel in situ when applied to the surgical margin, enabling liver-localized, sustained drug delivery and enhancing RT-induced NK cell activation. In murine models, this strategy successfully prevented intrahepatic recurrence and also triggered systemic CD8^+^ T cell responses, suppressing postoperative lung metastases. The hydrogel further exhibited rapid hemostatic properties in rat and porcine models [84].

A different approach involved the gelation of a CS-SH derivative induced by oxidized glutathione to encapsulate ultrasmall nanoparticles composed of oxidized starch (SNPs) (~10–40 nm). The resulting nanoassemblies were loaded with DOX and tested for the treatment of colon cancer both in vitro and in vivo. The larger CS-based nanoassembly served as a protective outer layer that enabled systemic circulation and tumor targeting, while the smaller SNPs, with their high DOX-loading capability (through imine interactions between aldehyde groups and unprotonated DOX), ensured deep tumor penetration. Once in the reductive tumor microenvironment, the disulfide bonds were cleaved, causing the nanogel to degrade and release the smaller SNPs for localized drug delivery. CS coating was found to significantly suppress tumor growth (20.0× vs. 11.1× for free and encapsulated DOX) [85].

A methoxy polyethylene glycol–CS conjugate (mPEG-CS) was proposed as a coating for the targeted delivery of a Platinum(IV) prodrug to ovarian and colon cancers. In one approach, a chlorin e6 (Ce6)-Pt(IV) prodrug and a polyaspartamide (PAS)-mPEG derivative were conjugated via 1-ethyl-3-(3-dimethylaminopropyl)carbodiimide (EDC) chemistry, coordinated with a gallic acid (GA)-Fe^3+^ complex, and finally coated with mPEG-CS to neutralize the positive charge of the nanosystem. In colon cancer, the acidic conditions of the tumor microenvironment and intracellular lysosomes induce the dissociation of the GA-Fe^3+^ complex from Ce6-Pt(IV), enabling photodynamic therapy through activation of Ce6-Pt(IV) upon red light irradiation. The subsequent ROS generation reduces Pt(IV) to Pt(II) complexes, thereby initiating chemotherapy. In addition, ferroptosis is triggered by the reduction in ferric ions by released GA, together with depletion of intracellular GSH, ultimately promoting macrophage polarization in cancer cells [86].

Similarly, the photothermal sensitizer IR-820 was conjugated to PEI along with a norcantharidin–platinum (NCTD-Pt(IV)) prodrug and coated with mPEG-CS. Once internalized by ovarian cancer cells, NIR irradiation induced nanoplatform disassembly and reduction in NCTD-Pt(IV), thereby enabling the cytotoxic action of NCTD and Pt(II). At the same time, the photothermal effect increased the intracellular concentration of calcium ions, disrupting the circadian clock of cancer cells and rendering them more sensitive to chemotherapeutics [87].

### 4.2. Self-Assembling CS Derivatives

Besides being used as a coating element, a different approach involved the use of CS as a structural component in NP architecture. In particular, the insertion of lipophilic moieties into the CS structure allowed the development of amphiphilic CS derivatives capable of self-assembly in aqueous environments. Different strategies have been proposed to achieve this goal, mainly involving the use of cholesterol and/or fatty acids as derivatizing agents (Table 4).

Cholesterol (Chl) has been employed in nanoparticle design either for its fundamental role as a component of cell membranes, which facilitates drug permeability, or for its elevated serum levels in cancer tissues that sustain the uncontrolled growth of cancer cells.

Mono- and dual-responsive DOX delivery systems were obtained through the self-assembly of CS-Chl conjugates synthesized using formate and succinate/thioketal linkers, respectively. The core concept of these approaches lies in combining the active targeting ability of CS with the distinctive features of tumor tissues: formate and succinate groups are cleavable under acidic pH, while thioketal spacers are sensitive to the oxidative environment. Both nanosystems exhibited high anticancer activity in vitro and in vivo, with the mono-responsive carrier achieving a breast cancer inhibition rate of 76% compared to 63% for free DOX [88], and the dual-responsive nanosystem reducing the DOX IC_50_ from 2.074 to 0.912 μg mL^−1^ [89].

Deoxycholic acid (DChA) was also employed as the hydrophobic moiety of CS amphiphiles for DOX-based breast cancer treatment in both in vitro and in vivo models. Dual-targeting strategies were proposed, with the CDVEWVDVS peptide and the AS1411 aptamer serving as targeting elements for P-selectin and nucleolin, respectively. The CS-DChA conjugate was synthesized using cys as a redox-responsive linker to trigger drug release within the tumor microenvironment. As a result, the peptide-containing carrier exhibited greater efficacy (*p* < 0.05) in CD44-overexpressing MDA-MB-231 cells compared to non-triple-negative MCF-7 cells [90], while the aptamer-based material displayed high cell invasion inhibition (77% vs. 25% for free DOX) [91]

Maleimide (MAL) was incorporated to enhance cellular uptake and extend the blood circulation of the CS-DChA–based Docetaxel (DTX) nanocarrier. This system operates by exploiting the transient binding of MAL with thiol groups in blood components and cell membranes, thereby boosting the CD44-targeting ability of CS. Specifically, PEG-NH_2_ was used as a linker between CS and MAL residues, while protonation of the carboxylate ions in the CS backbone at the tumor site modulated drug–polymer interactions, leading to enhanced drug release [92].

**Figure 5:**
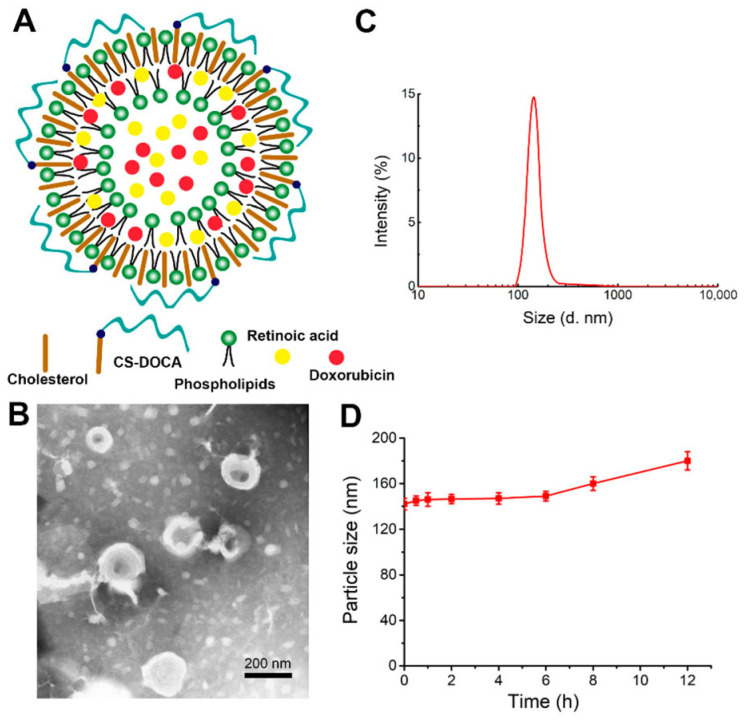
CS-modified liposomes for the co-delivery of DOX and ReA to breast cancer cells. (**A**) hypothesized structure; (**B**) Transmission electron micrograph; (**C**) mean hydrodynamic diameter; (**D**) stability overtime. Reproduced from [94].

CS-DChA was combined with lipid components for the fabrication of lipid NPs or liposomes (hydrodynamic diameter of around 100 nm and high colloidal stability ovetime) via thin-film hydration, proposed as a strategy to combine the cytotoxic activity of DOX with the anti-metastatic activity of retinoic acid (ReA), which interferes with Golgi apparatus function. DOX and ReA were co-loaded through the formation of an electrostatic complex with high efficiency, enabling sustained release for up to 96 h and demonstrating significant anticancer activity against liver [93], melanoma, and breast cancers (Figure 5) [94].

A TPGS/CS–cholic acid (ChA) micellar structure was designed for the delivery of an Elesclomol (ES)–Cu complex, capable of inducing mitochondrial oxidative stress and copper-dependent cell death. ES moves cupric ions from the extracellular matrix into the cytoplasm, where it triggers proteotoxic stress and subsequent cuproptosis. Specifically, a CS-ChA/CuSO_4_ aqueous solution was used as the hydrating medium for an ES/TPGS film. After optimization to ensure serum stability, the NPs were shown to overcome MDR and induce cell death without impairing efflux transporter activity, which is desirable since P-gp activity protects healthy cells and tissues from PTX toxicity. Moreover, the release of oxidized mitochondrial DNA triggered an immune response via multiple pathways [95].

The stimulation of the immune response was achieved by coating cationic liposomes with a mixture of CS-glycocholic acid (CS-GCA) and CS-mannose (CS-MN) derivatives. Specifically, negatively charged OVA and polyinosine–polycytidylic acid (PIC), acting as an antigen and a cancer-specific adjuvant, respectively, were electrostatically loaded onto the liposome surface and further coated with the CS derivatives. Repeated oral administration of the nanoplatform increased the populations of CD3^+^ CD8^+^ T cells, CD44^high^ CD62L^low^ memory T cells, and CD11b^+^ CD27^+^ natural killer cells in the blood, while reducing serum levels of CD4^+^ CD25^+^ Foxp3^+^ regulatory T cells, thereby completely preventing melanoma development in melanoma-bearing mice [96].

**Figure 6:**
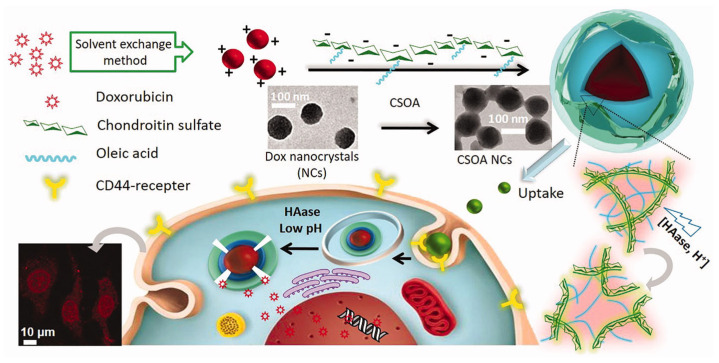
Schematic representation of targeted DOX delivery system by CS coating of DOX nanocrystals. Reproduced from [97].

Amphiphilic CS derivatives obtained through conjugation with fatty acid derivatives (i.e., oleic acid, OA, and Fmoc-8-amino-3,6-dioxaoctanoic acid, Fmoc-AEEA) or 3-indoleacetic acid (IAA) were proposed as coating elements for DOX and PTX nanocrystals, improving the water solubility of the drug crystals and consistently enhancing their anticancer activity against different solid tumors. DOX-loaded NPs (based on CS-OA, ~100 nm) were more effective than conventional DOX micelles prepared with PEG derivatives, with drug release triggered by pH and hyaluronidase activity (Figure 6) [97].

PTX nanocarriers (based on CS-Fmoc-AEEA [98] or CS-IAA [99], ~200 nm) effectively targeted the Golgi apparatus and induced ROS production both in vitro and in vivo. Furthermore, combined treatment with CS-IAA nanoplatforms and αPD-L1 antibodies prolonged survival in in vivo studies.

The conjugation of CS with octadecylamine (ODA) was explored for the preparation of CUR nanogel (~60 nm), achieving prolonged release (over 70 h) and an increased proportion of cells in the sub-G1 phase [100]. In similar approaches, CS was lipidized via conjugation with palmitic acid (PA) and arachidonic acid (AA) through a cys linker to prepare redox-responsive nanocarriers. In detail, the self-assembly of CS-ss-PA was employed for the vectorization of Tamoxifen (TMX) to breast cancer cells with high efficiency, achieving a 60% improvement in survival compared to the control group [101]. Nanomedicine-driven ferroptosis was the core concept of CS-ss-AA–based nanosystems. CS-AA was self-assembled in PPI aqueous solution and decorated with Fe^3+^ ions through coordination with CS hydroxyl groups. A dual effect of exogenous and endogenous iron delivery was observed: the exogenous iron corresponded to the loaded Fe^3+^, while PPI promoted endogenous iron release by inducing ferritinophagy and ferritin degradation. AA residues served as substrates for lipid peroxidation, further enhancing oxidative stress [102].

Alpha-tocopherol succinate (TOS) was also employed as a CS lipidizing agent to obtain self-assembling micelles capable of delivering DOX to melanoma cells both in vitro and in vivo. This system effectively inhibited the metastatic process through the combined cytotoxic effect of DOX and the ability of TOS to inhibit matrix metalloproteinase-9 [103]. In a similar approach, the use of cys as a linker in the TOS–CS conjugation enabled the development of a highly effective DTX delivery vehicle. Treatment with redox-sensitive NPs resulted in superior antitumor efficacy, as evidenced by the necrosis rate (5%), late apoptosis rate (2%), early apoptosis rate (42%), and percentage of normal cells (52%), which were more favorable than those obtained with non-redox-sensitive NPs prepared using adipic dihydrazide instead of cys. In the latter case, the corresponding values were 2% (necrosis rate), 6% (late apoptosis rate), 34% (early apoptosis rate), and 58% (percentage of normal cells) [104].

A 1,2-distearoyl-sn-glycero-3-phosphoethanolamine (DSPE)-PEG-CS conjugate was proposed as an amphiphilic component for the preparation of liposomal and micellar nanosystems, designed for co-administration in a three-drug combination treatment of breast cancer. In this strategy, DOX was employed as a cytotoxic agent (loaded into micelles), while Mifepristone (Ru486) and Ber were incorporated into pH-responsive liposomes as metastasis inhibitors, achieving high tumor inhibitory rates and significant reduction in tumor volume, ECM deposition and tumor angiogenesis [105].

Another class of amphiphilic CS derivative consists of ZN-CS conjugates, which were used to prepare micellar systems for the treatment of breast cancer. In detail, etoposide (ETP) and all-trans retinoic acid (ATRA) were employed as bioactive agents for topoisomerase II inhibition and apoptosis induction/immune system activation, respectively. The micelles were further stabilized by Ca^2+^ crosslinking, achieving high synergistic antitumor efficacy (combination index: 0.8) [106].

As an extension of this concept, the same authors demonstrated that a Zein–sulphasalazine–CS conjugate (ZN-SFZ-CS) was able to potentiate the anticancer effect of Cls. In this nanoplatform, ZN acted as a Cls-loading promoter, CS as the targeting element, and SFZ as an inhibitor of the pro-inflammatory NF-κB pathway and inducer of apoptosis in cancer cells. Notably, SFZ also reduced intracellular GSH levels, thereby preventing Cls from reductive inactivation. Furthermore, a dual-targeting effect was achieved by incorporating superparamagnetic iron oxide nanoparticles (SPIONs) into the micellar core. This system enabled prolonged Cls release, with an SFZ/Cls combination index of 0.7. The added value of SPION loading was highlighted by the further reduction in IC_50_ upon application of an external magnetic field [107].

## 5. CS-Drug Conjugates

The conjugation of anticancer drugs to CS with the obtainment of polymeric prodrugs, is a valuable approach for the synthesis of effective delivery vehicles. Compared with their low-molecular-weight counterparts, polymeric prodrugs offer several advantages: (1) improved water solubility of poorly soluble or insoluble drugs, enhancing bioavailability; (2) protection of the drug from deactivation, thus maintaining its activity during circulation, transport to organs or tissues, and intracellular delivery; (3) improved pharmacokinetic properties; (4) reduced antigenicity, resulting in a milder immune response; (5) potential for passive or active targeting of the drug to its specific site of action; and (6) the ability to form advanced drug delivery systems that, besides the drug and polymer carrier, may include additional active components enhancing the drug’s specific activity [108]. The main examples of applying this concept to CS NPs are collected in Table 5.

Environmental-responsive micellar systems were developed by conjugating anticancer drugs to CS through suitable cleavable spacers. CS-DTX and CS-flurbiprofen (CS-FBP) conjugates were co-assembled into monodisperse micelles with an average diameter of approximately 200 nm, thereby combining the complementary anticancer effects of DTX and COX-2 inhibition in promoting cancer cell apoptosis. DTX acts by binding to tubulin, stabilizing microtubules, and disrupting mitosis, whereas FBP suppresses prostaglandin E_2_ production, thereby reducing cell proliferation and angiogenesis. Specifically, DTX and FBP were linked to CS via redox-responsive (cys) and pH-sensitive (EDC-mediated) bonds, respectively, achieving a synergistic effect on breast cancer models [109]. In a similar strategy, a redox-responsive CS-Celecoxib (CS-CXB) conjugate was self-assembled to encapsulate and enhance the anticancer efficacy of Camptothecin (CPT) against colon cancer. This formulation also contributed to reducing oxidative stress and improving the inflammatory microenvironment in murine models, as evidenced by decreased TNF-α and IL-6 levels [110].

A self-assembling CS-DOX conjugate, synthesized with adipic dihydrazide (ADH) as a pH-responsive linker, was developed for the combined DOX/berberine (Ber) treatment of breast cancer. By simultaneously inducing apoptosis through DOX and remodeling the tumor microenvironment via Ber, the nanoplatform achieved a marked tumor growth inhibition of approximately 95% [111].

In a different approach, chlorambucil (Chl) was conjugated to CS via amide bonding to obtain enzyme-responsive nanoparticles (NPs), which were further endowed with redox sensitivity by co-assembling a CS–lipoic acid (CS–LA) conjugate. In vitro viability studies on MDA-MB-231 breast cancer cells demonstrated a marked enhancement of the anticancer activity of Chl compared to the free drug (cell death of 89% vs. 38%), which further increased to 95% upon DOX encapsulation within the NPs [112].

**Figure 7:**
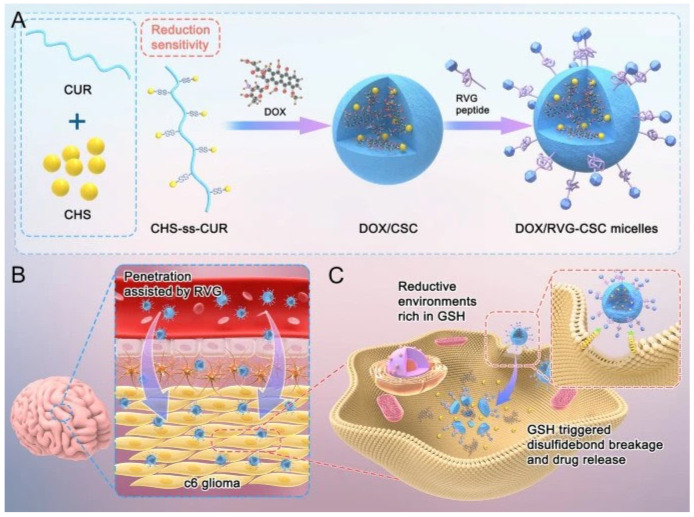
(**A**) Schematic representation of CS-ss-CUR micelles formation. (**B**) BBB Penetration. (**C**) Active tumor cell entry and glutathione triggered drug release. Reproduced from [114].

The conjugation of CS to rosmarinic acid (RA) and CUR via a cys linker enabled the formation of micelles for the selective, redox-sensitive delivery of doxorubicin (DOX) to colon and brain cancers, respectively. Specifically, CS-ss-RA was co-assembled with DSPE-PEG to obtain micelles with a mean diameter of 190 nm, which achieved optimal tumor suppression (600 and 230 mm^3^ for free and encapsulated DOX, respectively) after 19 days of treatment [113]. Similarly, DOX was encapsulated in CS-ss-CUR micelles, which were further functionalized with the neurotropic rabies virus-derived polypeptide (RVG) and evaluated for glioma treatment. By combining the CD44-targeting capability of CS, the redox sensitivity conferred by cys, the ability of RVG to enhance blood–brain barrier penetration, and the P-gp inhibition effect of CUR, the micelles effectively suppressed glioma growth while causing negligible adverse effects and preserving brain function (Figure 7) [114]. 

A multifunctional CS conjugate was developed using ADH as a pH-responsive linker for cinnamaldehyde (CA) and CUR as a ligand for TPP. Under acidic conditions, NPs destabilization and lysosomal escape enabled CUR to target and damage mitochondria through TPP-mediated localization. CA acted synergistically with CUR to generate high levels of ROS and deplete intracellular GSH, thereby disrupting redox homeostasis and inducing ferroptosis via downregulation of GPX4 and xCT expression. The onset of ferroptosis further triggered immunogenic cell death, promoting the secretion of IFN-γ, TNF-α, and IL-6 [115].

Taking advantage by the high yield of singlet oxygen produced upon laser irradiation within the 660–670 nm, CS-Chlorin e6 (Ce6) conjugate were proposed for combined photodynamic and DOX/PTX based chemotherapy treatment of breast cancer, with the use of cys as a linker allowing redox-responsive behavior. In the first case, NPs with diameter of 270 nm possessed superior antitumor activity against 4T1 and MDA-MB-231 cells [116], while the co-loading of QR into PTX nanocarriers was proposed as a strategy to overcome MDR by P-gp inhibition [117].

A CS–ICG conjugate was employed as a targeted delivery vehicle for erastin (ERS), a potent ferroptosis-inducing system x_c_^−^ inhibitor with significant clinical limitations due to its poor physicochemical and pharmacokinetic properties. NPs, with a mean diameter of 190 nm, enabled sustained ERS release over 120 h and induced G1-phase cell cycle arrest, thereby inhibiting cancer cell proliferation predominantly through a non-ferroptotic mechanism. Furthermore, ICG enhanced cytotoxicity upon laser activation, resulting in a tumor reduction 2.5-fold greater than that achieved with free ERS. Remarkably, NPs significantly improved tumor-specific accumulation of ERS (1.9-fold higher than free drug) while markedly reducing off-target deposition in the lungs and spleen by 203-fold and 19.1-fold, respectively [118].

In a related strategy, an ICG analogue (IR806) was conjugated to CS via a cys linker to enable synergistic sonodynamic and phototherapy (both photothermal and photodynamic) against localized prostate cancer. The resulting NPs exhibited substantially enhanced water solubility, dramatically reduced dark toxicity, and excellent biocompatibility compared to the free sensitizers. They also demonstrated CD44 receptor-mediated endocytosis and subcellular mitochondrial targeting, along with dual redox- and hyaluronidase-responsiveness, collectively enhancing cellular uptake and therapeutic efficacy. Upon dual sono/photoirradiation, NPs generated effective hyperthermia and high levels of ROS, achieving synergistic antitumor activity via complementary mechanisms of sonodynamic and phototherapy (combination index: 0.7) [119].

Rhein (Rh), a naturally occurring anthraquinone with various pharmacological activities, including ROS generating activity, was also conjugated to CS for a sonodynamic treatment of melanoma and lung cancers. In the first case, the conjugation of CS to Rh improved solubility, tumor-targeting ability, and antitumor activity compared with free Rh. Furthermore, the linkage to perfluorocarbon (PFC) enabled NPs to carry and deliver oxygen to B16F10 melanoma cells, resulting in higher ROS production and stronger tumor inhibition both in vitro and in vivo. Notably, Rh can also directly and gradually generate ROS through the c-Jun N-terminal kinase (JNK)/Jun/caspase-3 signaling pathway, increasing ROS levels in deeper tissues not directly exposed to ultrasound energy. In addition, Rh inhibits tumor growth by activating apoptosis-related proteins, including caspase-3, BCL-2, and BAX, and by suppressing tumor angiogenesis. When loaded with DTX, NPs further promoted immunogenic cell death, and enhanced antitumor immune responses as evidenced by the significant increase in tumor-infiltrating CD4^+^ and CD8^+^ T cell populations [120]. 

**Figure 8:**
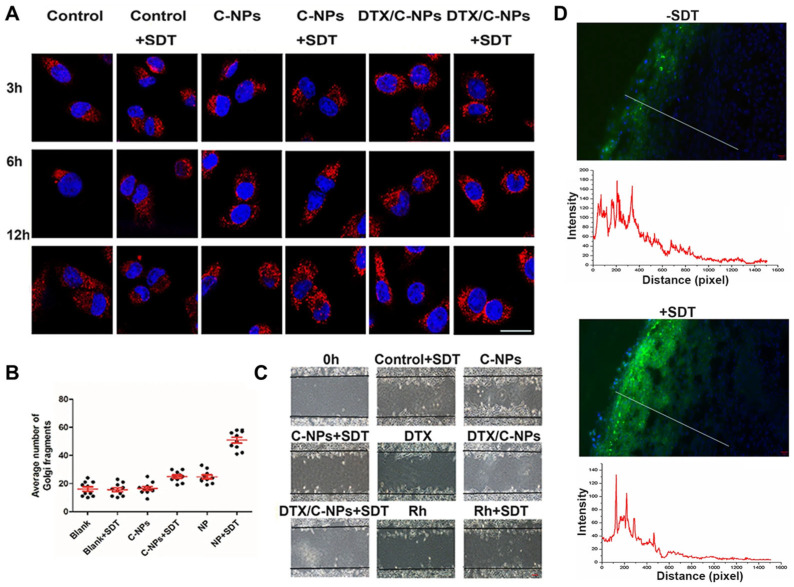
(**A**) Integrity of Golgi apparatus structure incubated with different preparations with/without SDT treatment. (**B**) Fragmented quantitation in Golgi apparatus for A549 cells after treatments. (**C**) Wound healing ability of A549 cells incubated in the absence and presence of SDT for 24 h. (**D**) Fluorescence distribution and intensity of NPs in A549 solid tumor with/without ultrasound treatment. Reproduced from [121].

For lung cancer treatment, DTX was loaded on NPs obtained by the self-assembly of CS-ADH-Rh-LA conjugate, where the LA ability to form intermolecular disulfide bonds was exploited to confer combined redox/ultrasound-responsive properties. NPs selectively targeted subcellular organelles, including the Golgi apparatus and mitochondria, causing structural damage, while encapsulated DTX further disrupted microtubule morphology and blocked the mitotic cycle of A549 cancer cells, amplifying the antitumor effect. Ultrasound (SDT) treatment enhanced tumor tissue penetration of NPs, enabling deeper distribution and more effective tumor suppression (Figure 8). The nanoplatform also modulated the tumor immune microenvironment by reducing M2-type macrophages and promoting M1-type macrophages, while concurrently inhibiting tumor angiogenesis [121].

## 6. Conclusions and Outlook

CS-based NPs have emerged as a promising class of smart nanomedicines for cancer therapy, offering a unique combination of biological activities (e.g., anti-inflammatory, antioxidant, anti-thrombotic, and neuroprotective properties) and physicochemical versatility. Their intrinsic anti-angiogenic and antimetastatic properties, along with immune-modulatory effects, make them attractive candidates for targeting tumor cells while minimizing off-target toxicity. CS NPs can exploit receptor-mediated endocytosis for tumor-specific delivery, and their physicochemical properties, such as enhanced stability, reduced immune recognition, and favorable biodistribution, distinguish them from conventional nanocarriers. These characteristics, combined with their potential for theranostic applications, enable the simultaneous delivery of therapeutic agents and imaging molecules, supporting both cancer treatment and diagnosis.

Despite their promise, clinical translation of CS NPs remains limited. The primary barrier to commercialization, as with other nanoparticle systems, is regulatory approval. Manufacturers must demonstrate both short- and long-term safety and efficacy in humans, a process that is highly labor-intensive and time-consuming. The lack of standardized guidelines often complicates this process further. Additionally, large-scale production poses challenge due to the inherent complexity of multi-component NPs. Conventional synthesis methods frequently yield variable particle size, low drug loading, and inconsistent reproducibility, limiting their feasibility for clinical applications. Emerging techniques such as microemulsion, nanoprecipitation, microfluidics, and self-assembly offer scalable, reproducible, and cost-effective alternatives for manufacturing CS NPs, providing tighter control over size distribution, encapsulation efficiency, and stability. In addition, CS faces significant regulatory challenges due to its biological origin, variability in purity, and its dual classification as both a dietary supplement (subject to minimal regulation) and a potential drug or biomaterial (requiring strict regulatory oversight). Major issues include risks of contamination (e.g., viruses, allergens), structural inconsistency arising from differences in source and processing methods, and questions regarding efficacy, particularly when distinguishing between nutraceutical-grade products, which often exhibit high variability, and pharmaceutical-grade CS, which requires well-defined characteristics.

A critical factor in successful translation is the accurate prediction of in vivo behavior. Traditional in vitro models often fail to replicate the complex tumor microenvironment, contributing to discrepancies between laboratory results and clinical outcomes. Integrating organoids, co-culture systems, and organ-on-a-chip platforms can better mimic physiological conditions, providing insights into nanoparticle biodistribution, cellular uptake, and drug release. Patient-derived xenografts, genetically engineered mouse models, and humanized mice offer improved representations of tumor heterogeneity and metastasis, yet no model fully captures human malignancy. CS NPs, with their capacity to evade the mononuclear phagocyte system and penetrate complex tumor microenvironments, are particularly well-suited to address these challenges. Multi-pathway-targeting CS formulations further enhance therapeutic efficacy by overcoming barriers such as intratumoral pressure, multidrug resistance, and endosomal entrapment.

Recent research has highlighted the importance of subcellular organelle targeting in enhancing nanoparticle efficacy. Interactions among organelles, including the endoplasmic reticulum, mitochondria, Golgi apparatus, and lysosomes, play essential roles in maintaining cellular homeostasis. CS NPs can be engineered to disrupt these interactions selectively, inducing ferroptosis, impairing mitochondrial function, or interfering with ER–Golgi signaling to promote apoptosis. Multi-organelle-targeted formulations, pH-responsive biomineralized CS liposomes, and synthetic peptides with ER- or Golgi-specific affinity have demonstrated significant antitumor effects in preclinical models.

Modifying nanoparticle charge, size, and surface chemistry can improve endocytic uptake and prevent lysosomal entrapment. Functionalization such as polymeric shells or triphenylphosphonium conjugation have further extended circulation time and tumor accumulation, paralleling strategies successfully employed in other nanoparticle systems.

Artificial intelligence (AI) and computational modeling are playing an increasingly vital role in optimizing CS-based nanoparticle design. Traditional computational tools such as molecular docking, molecular dynamics, and quantitative structure–activity relationships (QSAR) have long supported nanoparticle formulation, but their integration with AI, machine learning, deep learning, and big data analytics now provides far more powerful predictive capabilities. These integrated approaches enable accurate forecasting of drug loading efficiency, release kinetics, cellular interactions, and systemic distribution. In addition, AI-driven algorithms can anticipate patient-specific responses, refine dosing strategies, and minimize empirical trial-and-error processes, thereby accelerating the path toward clinical translation.

Looking ahead, CS-based NPs hold significant promise for the development of patient-friendly, controlled, and potentially self-administered cancer therapies, especially as AI-supported design continues to demonstrate its transformative potential. Machine learning tools are increasingly able to uncover hidden correlations within large datasets, predict formulation performance, and guide the rational engineering of delivery systems, allowing researchers to explore expansive formulation landscapes with unprecedented precision. When applied to nanoparticle development, AI expedites the identification of innovative CS-based architectures, optimizes their biodistribution and cellular uptake, and improves understanding of their interactions with the immune system, reducing reliance on labor-intensive experimental screening. By integrating AI-guided design with organelle-targeted delivery, modulation of multiple therapeutic pathways, and strategic engagement of immune mechanisms, CS nanomedicines may overcome many of the limitations associated with conventional cancer treatments. This convergence of AI and nanotechnology positions CS-based NPs as a powerful platform capable of delivering safer, more effective, and personalized anticancer therapies that transform technological innovation into real clinical benefit.

## Figures and Tables

**Table 1:** CS as a coating for organic nanocarriers.

Composition		Preparation	Drug	Cancer Model			Results	Ref.
CS Derivative	Other Components			Tissue	In Vitro	In Vivo		
CS	TMCH	Complexation	TMCH	Ovary	SKOV-3 OVISE	---	CD44 targeting	[41]
CS/DOX	BSA	Electrostatic interaction	DOX	Breast	4T1	4T1 orthotopic	CD44 targeting Enhanced antitumor	[42]
CS	β-CyD-PEI	Complexation	PTX siRNA (siCD146)	Breast	MDA-MB-231	---	CD44 targeting Synergism Antimetastatic proapoptotic	[43]
CS	CH	Electrostatic interactions	CUR	Cervix	HeLa	---	Synergism	[44]
				Colon	HT29			
				Prostate	PC3			
CS	CH	Complexation	CUR-Ag	Colon	Caco-2	---	Synergism Photodynamic	[45]
CS	TRL/PC/lPC/ChO/ Chl TPGS Lf	Solvent- emulsification layer-by-layer	Fis	Breast	MDA-MB-231	EAT mice	CD44 targeting	[46]
CS	CMP/P407/P188 Lf	Ultrasonication Layer-by-layer	PTS	Breast	MDA-MB-231	MDA-MB-231 Orthotopic	Phytotherapy	[47]
CS	CH CQD	Layer-by-layer assembly (ionic coating)	DOX DTX	Bone	U2OS Soas-2	---	Prolonged release Synergism	[48]
CS	FMP-NaCas	Self-assembly Electrostatic interaction	CUR	Liver	HepG2 L-O2	---	CD44 targeting	[49]
CS	ZN	Solvent displacement	DTX	Prostate	PC-3	PC-3 xenograft	CD44 targeting Redox/pH responsive release No systemic toxicity	[50]
CS	ZN	Solvent displacement	TFM	Breast	MCF-7 MDA-MB-231	---	CD44 targeting pH responsive release Synergism	[51]
CS	MAL-PEG-ss-PLA	CS as coating	DOX	Breast	4T1	4T1-bearing	CD44 targeting pH/Redox responsive release ROS generation Synergism	[52]
CS	TPP-PEG-ss-PLA	Self-assembly	DOX	Liver	HepG2	H22 bearing	CD44 targeting Redox/pH-responsive Mitochondrial targeting	[53]
CS-FA	TPP-TPGS-PLGA	Coating	Cls	Breast	4T1	4T1 Orthotopic	Mitochondrial targeting	[54]
CS	DOTAP/PC/Chol TPGS BSA	Thin film hydration	PTX	Breast	MCF-7/MDR	MCF-7/MDR Xenograft	CD44 targeting MDR reversal	[55]
CS	HSPC Chol	Thin layer Hydration	Ber Mag	Lung	A549	A549 Orthotopic	CD44 targeting Prolonged circulation Synergism	[56]
CS	BD/hemin	Self-assembly Electrostatic interaction	Ber Hemin	Breast	MDA-MB-231	MDA-MB-231 Orthotopic	CD44 targeting	[57]
CS	PEI-OA	Thin layer hydration	CQ PTX OVA ATZ	Breast	4T1	4T1 Orthotopic mice	CD44 targeting Autophagy inhibition Tumor vaccine	[58]
				Cervix	HeLa	---		
CS	PEI-OA LNA-PEG-OCT	CS as coating	RNP PTX	Liver	HepG2	HepG2- bearing	CD44 targeting Somatostatin receptor targeting Synergism	[59]
CS	LyP-1-PC	Electrostatic interaction	PTX CTS	Breast	4T1	4T1 Orthotopic	CD44 targeting Immunotherapy	[60]
CS	PVP MM@P3	Nanoprecipitation	JQ1 CXB	Breast	4T1	4T1-bearing	CD44 targeting Synergism Immune response	[61]

β-CyD: β-cyclodextrin; ATZ: Atezolizumab; BD: 9-O-Octadecyl substituted berberine; Ber: Berberine; BSA: Bovine Serum Albumin; CH: Chitosan; Chl: Chlorambucil; ChO: Cholesteryl oleate; Chol: Cholesterol; Cls: Celastrol; CMP: Compritol^®^888; CQ: Chloroquine; CQD: Carbon Quantum Dot; CS: Chondroitin Sulfate; CTS: Cryptotanshinone; CUR: Curcumin; CXB: Celecoxib; DOTAP: 1,2-dioleoyl-3-trimethylammonium-propane; DOX: Doxorubicin; DTX: Docetaxel; EAT: Ehrlich ascites tumor; FA: Folic acid; Fis: Fisetin; FMP: Foxtail millet prolamin; HSPC: Hydrogenated soy phospholipid; JQ1: Bromodomain-containing protein 4 (BRD4) inhibitor; Lf: Lactoferrin; LNA: Linoleic acid; lPC: Lyso-phosphatidylcholine; LyP-1-PC: Peptide modified phosphatydilcholine liposome; Mag: Magnolol; MAL: Maleimine; MDR: Multi-drug resistance; MM: Melittin embedded macrophage membranes; NaCas: Sodium caseinate; OA: Oleic acid; OCT: Octreotide; OVA: Ovalbumin; P3: Branched Poly(β-amino Ester); P188: Poloxamers 188; P407: Poloxamers 407; PC: Phosphatidylcholine; PEG: Polyethylene glycol; PEI: Polyethyleneimine; PLA: Polylactide; PLGA: Poly(lactide-co-glycolide); PTS: Pterostilbene; PTX: Paclitaxel; PVP: Polyvinyl pyrrolidone; RNP: CRISPR/Cas9 ribonucleoprotein complex; siRNA: small interfering RNA; ss: Disulfide bond; TFM: Teriflunomide; TMCH: N,N,N-trimethyl chitosan; TPGS: D-α-tocopherol polyethylene glycol 1000 succinate; TPP: Triphenylphosphine; TRL: Triolein; ZN: Zein.

**Table 2:** CS as coating element for inorganic nanocarriers.

Composition		Preparation	Drug	Cancer Model			Results	Ref.
CS Derivative	Other Components			Tissue	In Vitro	In Vivo		
CS	CaCO_3_	Coating	ADM	Lung	A549 LLC	A549 bearing	CD44 Targeting Synergism	[67]
CS	PEG-HAP	EDC coupling	KAE	Colon	CT-26	CT-26-bearing	CD44 targeting pH responsive release Ca overload Pyroptosis Immunotherapy	[68]
CS	PEGS-HAP	EDC coupling	ATR	Colon	CT26	CT26 Orthotopic	CD44 targeting Ca overload Pyroptosis Immunotherapy	[69]
CS	MSNs-cys	Coating	PTX QR	Breast	MCF-7/ADR	MCF-7/ADR Xenograft	CD44 targeting Redox responsive release Synergism MDR reversal	[70]
CS	ZIF-8@A780	Sonication	IR780-ATO DOX	Breast	MCF-7 4T1	4T1 orthotopic	CD44 targeting Chemo-phototherapy MDR reversal	[71]
CS-ICG	SF-MnOx	water/oil emulsion	ICG	Colon	CT-26	CT-26 Orthotopic	CD44 targeting pH and redox responsive Immunotherapy	[72]
CS	EA-Cu	CS as coating	EA DOX	Breast	MCF-7/MCF-7ADR 4T1	4T1-bearing	CD44 targeting pH responsive release Synergism MDR reversal	[73]
CS	MOF-COD	EDC coupling	DOX COD	Breast	MCF-7/ADR	MCF-7/ADR Orthotopic	CD44 targeting Chemo sensitization	[74]
CS	Au	Multi-step process	---	Breast	MDA-MB-231 MDA-MB-468 T47D MCF7 SKBR3	---	CD44 targeting	[75]
				Brain	SH-SY5Y U87	---		
CS	Au	CS as coating	Au	Bone	MG63	---	CD44 targeting Mitochondrial damage	[76]
CS	c-sk Au	CS as coating	DOX	Breast	MDA-MB-231	---	Synergism	[77]
				Lung	A549			
				Stomach	AGS			
				Cervix	HeLa			
CS	Se	Dialysis	---	Cervix	HeLa	---	ROS generation Cell cycle arrest Apoptosis	[78]

A780: IR780 iodide; ADM: Adriamicin; ATO: Atovaquone; ATR: Atorvastatin; COD: Cholesterol oxidase; CS: Chondroitin Sulfate; c-sk: Yellow nose skate (Dipturus chilensis) cartilage extract; cys: Cystamine; DOX: Doxorubicin; EA: Ellagic Acid; EDC: 1-Etil-3-(3-dimetilamminopropil)carbodiimide; HAP: Hydroxyapatite; ICG: Indocyanine green; IR806: Carboxyl derivative of cyanine dye IR780; KAE: Kaempferol; MDR: Multi-drug resistance; MOF: Metal–organic framework; MSNs: Mesoporous silica nanoparticles; PEG: Polyethylene glycol; PEGS: Polyethylene glycol silane; PTX: Paclitaxel; QR: Quercetin; SF: Silk fibroin; ZIF-8: Zeolitic imidazolate framework-8.

**Table 3:** CS derivatives as coating elements of NPs.

Composition		Preparation	Drug	Cancer Model			Results	Ref.
CS Derivative	Other Components			Tissue	In Vitro	In Vivo		
OCS	MBGN-ss Gel	Schiff’s base bond network	MTX	Bone	UMR-106	osteosarcoma orthotopic	pH/GSH responsive Osteogenesis	[83]
OCS	PAAm Hf/TCPP MOF	Grounding Freeze-drying	3-MA ARL DNase I	Liver	HCC	HCC orthotopic	Radiotherapy Immune response Hemostasis	[84]
CS-SH	SNPs	Self-assembly (sonication)	DOX	Colon	CT26	CT26 Orthotopic	CD44 targeting Redox responsive release	[85]
mPEG-CS	PAS	Coating	Fe^3+^ Ce6-Pt(IV) GA	Colon	CT26	CT26 bearing	Photodynamic Ferroptosis	[86]
mPEG-CS	PEI	Coating	IR-820-NCTD-Pt(IV)	Ovary	HO-8910PM A2780 ID8	ID8 bearing	Chemosensitization Synergism DNA damage Apoptosis Photothermal	[87]

3-MA: 3-methyladenine; ARL: ARL67156 trisodium; Ce6: Chlorin e6; CS-SH: Thiol functionalized Chondroitin Sulfate; DNase I: NETs-lyase DNase I; DOX: Doxorubicin; GA: Gallic acid; Gel: Gelatin; GSH: Glutathione; Hf/TCPP: Hafniumions (Hf4+)/tetrakis (4-carboxyphenyl) porphyrin; MBGN: Mesoporous bioactive glass nanoparticles; MOF: Metal–organic framework; mPEG: Methoxy Polyethylene glycol; MTX: Methotrexate; NCTD-Pt(IV): Norcantharidin-platinum(IV) prodrug; OCS: Oxidized chondroitin sulfate; PAAm: Polyacrylamide; PAS: Polyaspartamide; PEI: Polyethyleneimine; SNPs: Starch nanoparticles.

**Table 4:** NPs prepared by self-assembling of CS derivatives.

Composition		Preparation	Drug	Cancer Model			Results	Ref.
CS Derivative	Other Components			Tissue	In Vitro	In Vivo		
CS-Chol	---	Self-Assembly	DOX	Breast	4T1 MCF-7 MDA-MB-231	4T1-bearing	pH responsive release Synergism CD44 targeting	[88]
CS-tk-Chol	---	Self-Assembly (dialysis)	DOX	Breast	4T1	4T1-bearing	CD44 targeting pH/redox responsive release Synergism	[89]
				Colon	CT-26	---		
t-Pep-CS-ss- DChA	---	Self-assembly (dialysis)	DOX	Breast	MCF-7 MDA-MB-231	MDA-MB-231 orthotopic	Redox-sensitive release CD44 targeting P-selectin targeting	[90]
AS1411-CS-ss- DChA	---	Self-assembly (dialysis)	DOX	Breast	4T1 MDA-MB-231	4T1 orthotopic	Redox-sensitive release Anti-metastatic	[91]
CS-DChA-PEG-MAL	---	Self-assembly	DTX	Breast	MCF-7	Sprague-Dawley	CD44 targeting pH responsive release Prolonged circulation	[92]
CS-DChA	Lct	thin-film hydration	DOX ReA	Liver	HSCs 7721	H22 Xenograft	CD44 targeting Anti-metastatic Synergism Prolonged release	[93]
CS-DChA	PL	thin film hydration	DOX ReA	Skin	B16F10	---	CD44 targeting Anti-metastatic Synergism Prolonged release	[94]
				Breast	4T1	4T1 orthotopic		
CS-ChA	ES−Cu TPGS	thin-film hydration	ES Cu	Prostate	DU145/DU145TXR PC3/PC3TXR	---	Immune response MDR reversal	[95]
				Lung	A549/A549TXR			
CS-MN/CS-GCA	DOTAP/DOPC	EDC coupling	OVA PIC	Breast	SK-BR-3	---	Immunotherapy	[96]
				Skin	B16F10	B16F10-bearing		
				Colon	CT26	---		
CS-OA	---	Solvent exchange/coating	DOX	Cervix	Hela	---	CD44 Targeting Enzyme responsive release	[97]
				Liver	HepG2			
CS-Fmoc-AEEA	---	Self-assembly	PTX	Pancreas	Panc02	Panc02 orthotopic	Improved PTX solubility CD44 targeting	[98]
				Skin	B16F10	---		
				Breast	4T1	---		
CS-IAA	---	solvent-anti-solvent (sonication)	PTX	Pancreas	Panc02	Panc02 orthotopic	CD44 targeting ROS generation synergism	[99]
CS-ODA	---	Self-assembly	CUR	Breast	MCF-7	---	Prolonged release CD44 targeting	[100]
CS-ss-PA	---	Self-assembly (sonication)	TMX	Breast	MCF-7 MDA-MB-231	MDA-MB-231 Orthotopic	CD44 targeting	[101]
CS-ss-AA	---	Self-Assembly (dialysis)	Fe PPI	Liver	Huh-7	Huh-7 xenograft	Ferroptosis Redox responsive release	[102]
CS-TOS	---	Self-assembly	DOX	Skin	B16F10	B16F10 orthotopic	CD44 Targeting pH responsive release Antimetastatic	[103]
CS-ss-TOS	---	Self-assembly	DTX	Skin	B16F10	B16F10 bearing	CD44 targetin Redox responsive release Reduced systemic toxicity	[104]
DSPE-PEG-CS	AIM-Chol Chol PL	thin-film dispersion	Ber Ru486	Breast	MCF-7	4T1-bearing	CD44 targeting Synergism	[105]
	PL GCA	ethanol injection	DOX					
ZN-CS	---	Self-assembly (Ca^2+^ crosslinker)	ETP ATRA	Breast	MCF-7	EAT bearing	CD44 Targeting	[106]
ZN-SFZ-CS	SPION	Self-assembly	Cls	Breast	MCF-7 MDA-MB-231	EAT bearing	CD44 Targeting Magnetic targeting Inhibition of NF-κB, VEGF and COX-2	[107]

AA: Arachidonic acid; AIM-Chol: (3-Aminopropyl) imidazole-Cholesteryl chloroformate; AS1411: DNA aptamer; ATRA: Trans-retinoic acid; Ber: Berberine; ChA: Cholic Acid; Chol: Cholesterol; Cls: Celastrol; CS: Chondroitin Sulfate; CUR: Curcumin; DChA: Deoxycholic Acid; DOPC: 1,2-dioleoyl-sn-glycerol-3-phosphocholine; DOTAP: 1,2-dioleoyl-3-trimethylammonium-propane; DOX: Doxorubicin; DSPE: 1,2-Distearoyl-sn-Glycero-3-Phosphoethanolamine; DTX: Docetaxel; EAT: Ehrlich ascites tumor; EDC: 1-Etil-3-(3-dimetilamminopropil)carbodiimide; ES: Elesclomol; ETP: Etoposide; Fmoc-AEEA: Fmoc-8-amino-3,6-dioxaoctanoic acid; GCA: Glycocholic Acid; IAA: 3-Indoleacetic Acid; Lct: Lecithin E80; MAL: Maleimine; MDR: Multi-drug resistance; MN: Mannose; OA: Oleic acid; ODA: Octadecylamine; OVA: Ovalbumin; PA: Palmitoyl; PEG: Polyethylene glycol; PIC: Polyinosine-polycytidylic acid; PL: Phospholipid; PPI: Polyphyllin I; PTX: Paclitaxel; ReA: Retinoic acid; Ru486: Mifepristone; SFZ: Sulfasalazine; SPION: Superparamagnetic iron oxide nanoparticles; ss: Disulfide bond; tk: Thioketal; TMX: Tamoxifen; TOS: D-α-tocopheryl succinate; t-Pep: Targeted CDVEWVDVS peptide; TPGS: D-α-tocopherol polyethylene glycol 1000 succinate; ZN: Zein.

**Table 5:** NPs composed of CS-based prodrugs.

Composition		Preparation	Drug	Cancer Model			Results	Ref.
CS Derivative	Other Components			Tissue	In Vitro	In Vivo		
CS-ss-DTX CS-FBP	---	Self-Assembly (sonication)	FBP DTX	Breast	MCF-7 MDA-MB-231	MFC-7 bearing	CD44 targeting pH/redox responsive release Synergism	[109]
CS-ss-CXB	---	Self-Assembly (dialysis)	CXB CPT	Colon	HT-29	HT-29-bearing	CD44 targeting Redox responsive release Lysosomal escape Synergism	[110]
CS-ADH-DOX	---	Self-Assembly (dialysis)	DOX Ber	Breast	MCF-7 4T1	4T1-bearing	CD44 targeting pH responsive release Synergism	[111]
CS-LA CS-Chl	---	Solvent evaporation	DOX Chl	Breast	MDA-MB-231	---	Redox responsive release Synergism	[112]
CS-ss-RA	DSPE-PEG	Self-Assembly	RA DOX	Colon	HT-29	HT-29 orthotopic	CD44 targeting Redox responsive release Anti-inflammation Synergism Lysosomal escape	[113]
CS-ss-CUR	RVG	Sonication	CUR DOX	Brain	C6 C6/ADR	C6/ADR Xenograft	Enhanced BBB permeability CD44 targeting Redox-responsive release MDR reversal Antimetastatic	[114]
CS-CA-CUR-TPP	---	Self-Assembly (stirring)	CUR CA	Lung	A549 H226	A549- bearing	CD44 targeting Ferroptosis pH responsive release Lysosomal escape Synergism	[115]
CS-ss-Ce6	---	Self-Assembly (dialysis)	Ce6 DOX	Breast	4T1 MDA-MB-231	4T1 xenograft	CD44 targeting Redox responsive release Synergism Photodynamic	[116]
CS-ss-Ce6	---	Self-assembly	Ce6 QR PTX	Breast	MCF-7 4T1	MCF-7/ADR xenograft	Photodynamic Redox responsive release Synergism MDR reversal Antimetastatic	[117]
CS-ICG	---	Self-Assembly (sonication)	ICG ERS	Liver	HCC SK-HEP Huh-7	SK-HEP-1 xenograft RIL-175 orthotopic	CD44 targeting Photothermal Photodynamic Synergism	[118]
CS-ss-IR806	---	Self-assembly	IR806	Prostate	PC-3	PC-3-bearing	Sonodynamic Photodynamic Photothermal Redox/enzyme responsive release CD44 targeting Mitochondria targeting	[119]
CS-ss-Rh-PFC	---	Self-assembly	Rh DTX	Skin	B16F10	B16F10-bearing	CD44 targeting Redox-responsive release Sonodynamic Immunotherapy	[120]
CS-ADH-Rh-LA	---	Sonication	Rh DTX	Lung	A549	A549 bearing	CD44 targeting Redox/ultrasound-sensitive release Sonodynamic Immunotherapy Anti-angiogenesis	[121]

ADH: Adipic dihydrazide; BBB: Blood-Brain-Barrier; Ber: Berberine; CA: Cinnamaldehyde; Ce6: Chlorin e6; CPT: Camptothecin; CS: Chondroitin Sulfate; CUR: Curcumin; CXB: Celecoxib; DOX: Doxorubicin; DSPE: 1,2-Distearoyl-sn-Glycero-3-Phosphoethanolamine; DTX: Docetaxel; ERS: Erastin; FBP: Flurbiprofen; ICG: Indocyanine green; IR806: Carboxyl derivative of cyanine dye IR780; LA: Lipoic Acid; MDR: Multi-drug resistance; PEG: Polyethylene glycol; PFC: Perfluorocarbon; PTX: Paclitaxel; QR: Quercetin; RA: Rosmarinic acid; Rh: Rhein; RVG: Rabies virus-derived polypeptide; ss: Disulfide bond; TPP: Triphenylphosphine.

## Data Availability

No new data were created or analyzed in this study.
